# Micronutrient deficiencies in ambulatory adults with cirrhosis

**DOI:** 10.1097/HC9.0000000000001032

**Published:** 2026-08-11

**Authors:** Melinda Wang, Kathy Pariani, Fawzy Barry, Dorothea Kent, Srilakshmi Seetharaman, Lisa Catalli, Jennifer C. Lai

**Affiliations:** 1 Department of Medicine, Division of Gastroenterology and Hepatology, University of California-San Francisco, San Francisco, California, USA; 2 School of Nursing, University of California, San Francisco, California, USA

**Keywords:** frailty, liver, mineral, nutrition, vitamin

## Abstract

**Background:**

Despite the well-established relationship between micronutrient deficiencies and cirrhosis, little is known of micronutrient deficiencies among ambulatory patients with cirrhosis awaiting liver transplant (LT). We aimed to characterize micronutrient deficiencies among patients with cirrhosis awaiting LT and assess their association with frailty, a complication with high morbidity and mortality.

**Methods:**

Ambulatory adult patients with cirrhosis awaiting LT from June 2024 to March 2025 were tested for micronutrient deficiencies in clinical practice. Multivariable modeling was used to assess the association between frailty and micronutrient deficiencies.

**Results:**

Of 275 patients, the median (IQR) age was 60 (51–66) years and 114 (42%) were women. Median MELD 3.0 (IQR) was 17 (12–22), and 64 (23%) were frail. Micronutrient deficiencies were common, especially of zinc, vitamin D/E/B12, and selenium. Compared to patients who were not frail, frail patients were more likely to have vitamin B12 deficiency (34% vs. 18%, *p*=0.01), vitamin E deficiency (36% vs. 20%, *p*=0.02), and selenium deficiency (28% vs. 13%, *p*=0.01). Patients with vitamin B12, vitamin E, or selenium deficiency had 2.56 (95% CI: 1.04–6.20), 2.55 (95% CI: 1.06–6.11), and 3.00 (95% CI: 1.15–7.68) increased odds of frailty on univariable analysis. When controlling for confounders, selenium deficiency alone remained independently associated with frailty (adjusted OR: 2.55, 95% CI: 1.18–5.54, *p*=0.02).

**Conclusions:**

Deficiencies of zinc, vitamin A/D/E/B12, and selenium are common in patients with cirrhosis awaiting LT and may represent a pragmatic panel of micronutrients to send in clinical practice. Selenium deficiency, in particular, was independently associated with frailty. Future studies should assess micronutrient repletion as a possible therapy in the multipronged approach to reversing frailty in patients with cirrhosis, as in the general population.

## Graphical abstract

**Figure:**
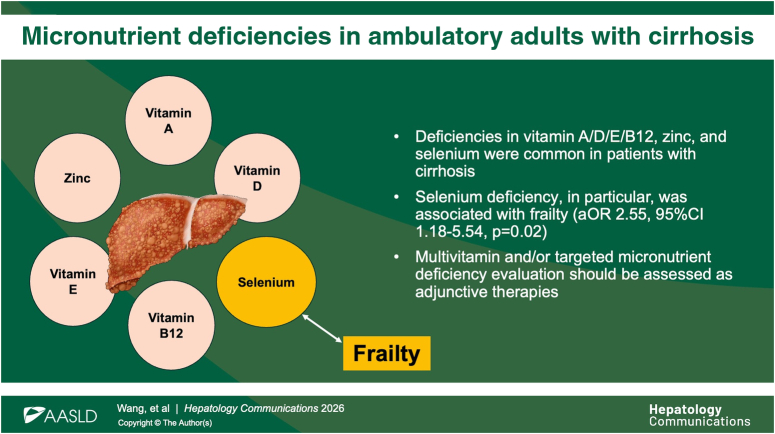

## INTRODUCTION

Cirrhosis is associated with alterations in nutrition intake and metabolism, leading to increased risk of protein-energy malnutrition/micronutrient deficiency and development of liver-related and non–liver-related morbidity and mortality.^1^,^2^,^3^ Liver disease leads to alterations in nutrition intake, metabolism, and the storage and activation of vitamins. Micronutrient deficiencies are well-established among hospitalized patients with decompensated cirrhosis.^1^,^2^,^4^ Given the widespread micronutrient deficiency associated with cirrhosis, empiric oral multivitamin supplementation has been recommended among patients with cirrhosis.^5^

Frailty is defined as a decreased physiological reserve and increased vulnerability to health stressors.^5^ Within geriatric literature, frailty itself is associated with micronutrient deficiencies among older adults.^6^,^7^ While many micronutrient deficiencies have been described among older adults with frailty, predominant micronutrient deficiencies include vitamin D and vitamin B12.^6^,^7^ Whether repletion of micronutrient deficiencies can prevent or reverse frailty within this population remains debated.

Among patients with cirrhosis, frailty leads to increased risk of decompensation, health care utilization, adverse post-transplant outcomes, waitlist mortality, and decreased health-related quality of life.^5^,^8^,^9^,^10^,^11^ The 2021 American Association for the Study of Liver Disease recommends routinely assessing for frailty among patients with cirrhosis at baseline and longitudinally.^5^ Given the significant impact of frailty among patients with cirrhosis awaiting liver transplantation, understanding the pattern of micronutrient deficiency and its association with frailty can elucidate potential micronutrient targets for frailty-mitigation intervention strategies. In this study, we aim to characterize micronutrient deficiencies among ambulatory adults with cirrhosis awaiting liver transplantation and identify specific micronutrient deficiencies associated with frailty.

## METHODS

All ambulatory adults with cirrhosis awaiting liver transplantation in initial evaluation for possible liver transplant listing at a single-center institution were tested for micronutrient deficiencies during routine protocolized clinical care from June 2024 to September 2025. Micronutrients tested as part of the panel were identified based on a literature review of commonly occurring micronutrient deficiencies identified in patients with cirrhosis and in older adults with frailty, reviewed by a nutrition-trained hepatologist and dietitian. Micronutrient deficiencies identified as commonly occurring included fat-soluble vitamins (vitamins B1, B6, B9, B12, and vitamin C) and minerals (zinc, selenium, and copper) due to impaired vitamin synthesis, activation and storage, altered protein and amino acid metabolism, inadequate oral intake, and losses due to commonly prescribed treatments such as lactulose and diuretics.^3^ Based on these micronutrient deficiencies, common micronutrient deficiencies among older adults who are frail include vitamin A, vitamins B6, B9, B12, vitamin D, and vitamin C.^6^ The final micronutrient panel was determined based on overlap between these 2 patient populations, laboratory test availability, and cost. The final micronutrients included the following: zinc, vitamin D, vitamin E, vitamin B12, selenium, vitamin A, vitamin B9, and vitamin B6. Thiamine deficiency was not included as thiamine deficiency is typically assessed by symptoms of deficiency, and patients within this cohort are often already treated or supplemented with thiamine, particularly within this cohort of patients with alcohol-associated liver disease who are at least 6 months from acute alcohol use. IRB# is 25-43788.^3^

The micronutrient panel was ordered during a required nutrition evaluation with a registered dietitian during initial pretransplant evaluation. Results of the micronutrient panel were subsequently communicated to patients by a registered dietitian, and any micronutrient deficiencies detected were repleted using a dietitian-driven micronutrient repletion protocol (Supplemental Figure S1, https://links.lww.com/HC9/C461). Sociodemographic and clinical variables were collected via chart review, including age, race, BMI, education, and marital status; cirrhosis etiology; MELD 3.0; dialysis status; and diagnoses of HCC, ascites, HE, hypertension, diabetes, coronary artery disease, stroke, and HIV. The primary outcome was frailty status, defined by liver frailty index ≥4.4 as defined previously as the cutoff for frailty in the pretransplant setting.^11^ The liver frailty index is a measure of physical frailty calculated using chair stands (time to complete 5 chair stands), balance testing (ability to balance side-by-side, semitandem, and tandem for 10 s), and grip strength (measured using a hand dynamometer of the dominant hand), validated among patients with cirrhosis. The liver frailty index was obtained at the clinical hepatologist's pretransplant evaluation on the same day as the nutrition pretransplant evaluation.

χ^2^ analysis and Wilcoxon rank sum tests were used to compare the frail versus not frail groups. Covariates were identified a priori based on clinical judgment and variables that were significant at a level *p*<0.05. Logistic regression was used to assess the association between frailty and micronutrient deficiencies identified as statistically significantly different in χ^2^ analysis. A 2-sided hypothesis test significance threshold of 0.05 was used in the study. Statistical analyses were performed using RStudio (Version 2025.05.0+496, Posit Software, PBC). Ethics approval for this study was provided by the University of California, San Francisco Institutional Review Board. All research was conducted in accordance with the Declarations of Helsinki and Istanbul. Written consent was waived for patients for whom only retrospective data were obtained.

## RESULTS

Of 275 total ambulatory adults with cirrhosis, 64 (23%) were frail, and 211 (77%) were not frail. Sociodemographic and clinical characteristics are summarized in Table 1. Frail and not frail patients did not significantly differ in age, gender, or race. Frail patients had significantly different cirrhosis etiology, lower hemoglobin and hematocrit levels, lower rates of HCC, and higher MELD scores (*p*<0.05) than not frail patients. A greater proportion of frail patients were also on dialysis and had ascites compared to not frail patients (*p*<0.05).

**TABLE 1:** Characteristics among patients with micronutrient testing by frailty status

Variable	Total n=275	Not frail n=211	Frail n=64	*p*
Sociodemographic characteristics				
Age	60 (51–66)	59 (53–66)	60 (50–65)	0.37
BMI	27.3 (24.6–31.7)	27.6 (25.0–31.8)	27.0 (23.3–30.8)	0.51
Women	114 (42)	83 (39)	31 (48)	0.25
Race				0.12
White	227 (83)	172 (82)	55 (86)	
Black	9 (3)	8 (4)	1 (2)	
Asian	33 (12)	28 (13)	5 (8)	
Other	3 (1)	1 (1)	2 (3)	
American Indian	1 (0.4)	0 (0)	1 (2)	
Native Hawaiian	1 (0.4)	1 (0.5)	0 (0)	
Hispanic	112 (41)	75 (36)	37 (58)	0.003
Education				0.23
<8th	49 (19)	33 (17)	16 (28)	
High school	97 (38)	74 (38)	23 (40)	
College	96 (38)	79 (40)	17 (29)	
Postgraduate	13 (5)	11 (6)	2 (4)	
Marital status				0.28
Committed	180 (66)	137 (66)	43 (68)	
Single	51 (19)	42 (20)	9 (14)	
Widowed	10 (4)	5 (2)	5 (8)	
Separated	28 (10)	22 (11)	6 (10)	
Unknown	2 (1)	2 (1)	0 (0)	
Clinical characteristics				
Etiology				0.004
MASLD	39 (15)	21 (11)	18 (30)	
EtOH	98 (39)	81 (42)	17 (28)	
Viral	38 (15)	33 (17)	5 (8)	
Autoimmune	14 (6)	12 (6)	2 (3)	
MetALD	2 (1)	1 (1)	1 (2)	
Multifactorial	64 (25)	46 (24)	17 (28)	
MELD 3.0	17 (12–22)	16 (11–20)	19 (15–23)	<0.001
Hemoglobin (g/dL)	11.4 (9.8–12.9)	11.6 (10.3–13.4)	10.4 (8.9–12.0)	<0.001
Hematocrit (%)	33.8 (29.7–38.0)	34.5 (31.0–39.2)	31.1 (27.5–36.1)	<0.001
MCV (fL)	93.0 (88.0–99.0)	98.0 (88.0–99.0)	94.0 (89.8–98.0)	0.94
Dialysis	15 (5)	7 (3)	8 (13)	0.01
HCC	65 (24)	57 (27)	8 (13)	0.03
Ascites	143 (61)	108 (58)	35 (76)	0.03
HE	160 (58)	116 (55)	44 (69)	0.07
Hypertension	132 (48)	94 (46)	38 (59)	0.05
Diabetes	94 (34)	64 (30)	30 (47)	0.02
Coronary artery disease	18 (7)	14 (7)	4 (6)	1.00
Stroke	4 (2)	2 (1)	2 (3)	0.23
HIV	5 (2)	4 (2)	1 (2)	1.00
Micronutrient deficiencies				
Vitamin A	191 (73)	143 (72)	48 (77)	0.48
B6	7 (3)	5 (3)	2(3)	0.67
B9	8 (3)	6 (3)	2 (3)	1.00
B12	58 (22)	36 (18)	22 (34)	0.01
Vitamin D	175 (64)	137 (66)	38 (59)	0.45
Vitamin E	65 (24)	42 (20)	23 (36)	0.02
Zinc	193 (71)	147 (71)	46 (72)	1.00
Selenium	46 (17)	28 (13)	18 (28)	0.01

Abbreviations: BMI, body mass index; EtOH, alcohol; MASLD, metabolic dysfunction–associated steatotic liver disease; MCV, mean corpuscular volume; MetALD, metabolic dysfunction- and alcohol-related steatotic liver disease.

**FIGURE 1:**
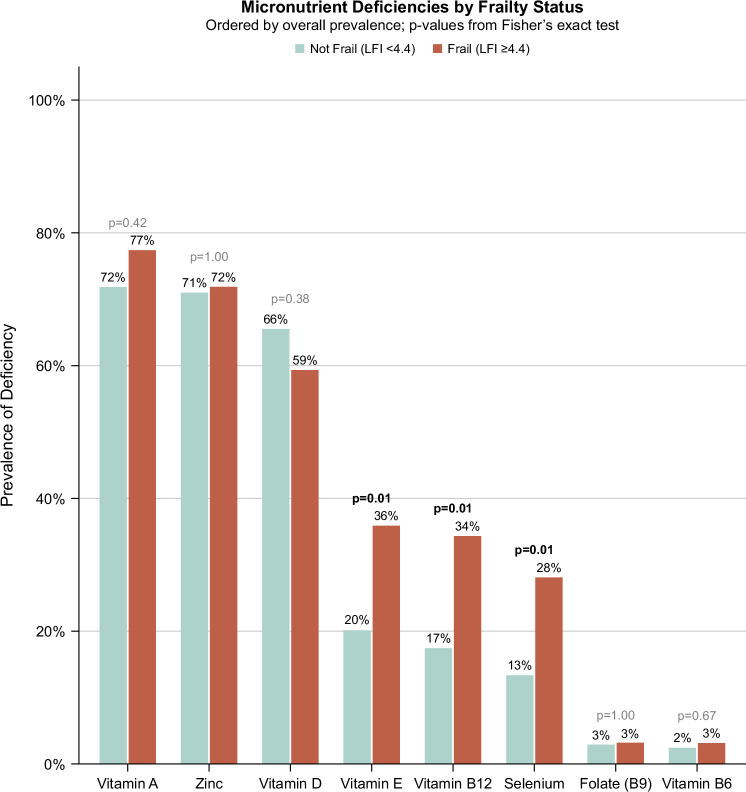
Micronutrient deficiencies among frail versus not frail patients. Abbreviation: LFI, liver frailty index.

Overall, ambulatory adults with cirrhosis in this cohort had high rates of vitamin A deficiency (73%), zinc deficiency (71%), vitamin D deficiency (64%), vitamin E deficiency (24%), vitamin B12 deficiency (22%), and selenium deficiency (17%) (Table 1). Adults with cirrhosis had low rates of vitamin B6 and B9 deficiency (3% respectively). Micronutrient deficiencies by cirrhosis etiology are described in Table 2. Zinc deficiency showed statistically significant variability among cirrhosis etiology groups. When evaluating patients with cholestatic versus noncholestatic cirrhosis, vitamin B6 deficiency was significantly higher among patients with cholestatic disease versus noncholestatic disease (2 [14%] vs. 4 [2%], *p*=0.04) (Supplemental Table S1, https://links.lww.com/HC9/C460). Compared to patients without ascites, patients with ascites had statistically significantly higher levels of vitamin A deficiency (111 [82%] vs. 49 [59%], *p*<0.001) and zinc deficiency (111 [79%] vs. 53 [60%], *p*=0.004) (Supplemental Table S2, https://links.lww.com/HC9/C460). Compared to not frail patients, frail patients had a greater proportion of vitamin B12 (34% vs. 18%, *p*=0.01), vitamin E (36% vs. 20%, *p*=0.02), and selenium deficiency (28% vs. 13%, *p*=0.01) (Table 1, Figure 1). Among patients with vitamin B12 deficiency, there was no significant difference in hemoglobin (10.7 [9.0–11.8] vs. 10.9 [9.6–11.8], *p*=0.35), hematocrit (31.1 [28.1–35.8] vs. 32.8 [29.6–36.3], *p*=0.24), and mean corpuscular volume (94.0 [91.2–98.7] vs. 91.0 [85.5–99.0], *p*=0.31) values between frail and not frail patients.

**TABLE 2:** Micronutrient deficiencies by cirrhosis etiology

	MASLD	EtOH	Viral	Autoimmune	Multifactorial	*p*
Vitamin A	27 (77)	75 (80)	25 (68)	10 (71)	45 (71)	0.56
Vitamin B6	1 (3)	2 (2)	0 (0)	2 (14)	1 (2)	0.11
Vitamin B9	0 (0)	4 (4)	0 (0)	0 (0)	2 (3)	0.70
Vitamin B12	12 (32)	20 (21)	7 (19)	3 (21)	13 (20)	0.67
Vitamin D	22 (56)	64 (66)	24 (63)	10 (71)	42 (65)	0.84
Vitamin E	10 (26)	25 (26)	6 (16)	6 (43)	16 (25)	0.38
Zinc	26 (67)	77 (80)	18 (47)	9 (69)	49 (75)	0.005
Selenium	7 (18)	19 (19)	4 (11)	3 (21)	11 (17)	0.78

On univariable analysis, vitamin B12, vitamin E, and selenium deficiency were associated with a greater than 2-fold odds of frailty (vitamin B12: OR 2.48, 95% CI: 1.31–4.63; vitamin E: OR 2.22, 95% CI: 1.19–4.08; selenium: OR 2.53, OR 1.27–4.95; *p*=0.01 for all, Table 3). When controlling for cirrhosis etiology, hemoglobin MELD 3.0 score, dialysis status, HCC, ascites, and HE, selenium deficiency (adjusted OR: 2.55, 95% CI: 1.18–5.54, *p*=0.02) but not vitamin B12 (adjusted OR: 2.02, 95% CI: 0.97–4.20, *p*=0.06) or vitamin E (adjusted OR: 1.87, 95% CI: 0.91–3.86, *p*=0.09) deficiency remained significantly associated with frailty. In the multivariable model, vitamin B12 and vitamin E deficiency remained significantly associated with frailty until hemoglobin was added to the model (data not shown).

**TABLE 3:** Univariable and multivariable analysis of micronutrient deficiency associated with frailty

	OR	95% CI	*p*	aOR	95% CI	*p*
Vitamin B12	2.47	1.31–4.63	0.01	2.02	0.97–4.20	0.06
MELD 3.0				1.03	0.97–1.08	0.37
Etiology						
MASLD				Ref	Ref	Ref
EtOH				0.23	0.10–0.51	<0.001
Viral				0.55	0.17–1.78	0.32
Autoimmune				0.27	0.08–0.09	0.05
Multifactorial				0.29	0.10–0.80	0.02
Dialysis				2.83	0.79–10.13	0.11
HCC				0.57	0.20–1.63	0.30
Ascites				1.87	1.08–3.23	0.02
HE				1.33	0.65–2.74	0.44
Hemoglobin				0.83	0.70–0.08	0.02
Vitamin E	2.22	1.19–4.08	0.01	1.87	0.91–3.86	0.09
MELD 3.0				1.03	0.96–1.08	0.58
Etiology						
MASLD				Ref	Ref	Ref
EtOH				0.23	0.10–0.51	<0.001
Viral				0.55	0.17–1.77	0.31
Autoimmune				0.25	0.07–0.92	0.04
Multifactorial				0.29	0.10–0.81	0.02
Dialysis				4.27	1.21–15.03	0.02
HCC				0.59	0.21–1.65	0.31
Ascites				1.93	1.11–3.32	0.02
HE				1.25	0.61–2.55	0.54
Hemoglobin				0.83	0.71–0.98	0.03
Selenium	2.53	1.27–4.95	0.01	2.55	1.18–5.54	0.02
MELD 3.0				1.02	0.96–1.08	0.51
Etiology						
MASLD				Ref	Ref	Ref
EtOH				0.22	0.10–0.48	<0.001
Viral				0.55	0.17–1.80	0.32
Autoimmune				0.27	0.07–0.98	0.05
Multifactorial				0.25	0.09–0.71	0.01
Dialysis				4.26	1.21–14.99	0.02
HCC				0.52	0.18–1.48	0.22
Ascites				1.83	1.06–3.17	0.03
HE				1.27	0.62–2.60	0.51
Hemoglobin				0.83	0.70–0.97	0.02

Abbreviations: aOR, adjusted odds ratio; EtOH, alcohol; MASLD, metabolic dysfunction–associated steatotic liver disease.

## DISCUSSION

In this study, we identified high rates of micronutrient deficiencies among ambulatory adults with cirrhosis awaiting liver transplantation and selenium deficiency specifically was associated with frailty even when controlling for other related factors. These findings provide a contemporary survey of micronutrient deficiency prevalence rates among a high-risk population of patients with cirrhosis and provide potential micronutrient targets that should be considered in future frailty-related interventions.

A myriad of micronutrient deficiencies have specifically been described among both older adults who are frail and hospitalized adults with cirrhosis.^1^,^2^,^6^,^7^ Llibre-Nieto et al^1^ found that hospitalized patients with acute decompensation of cirrhosis had a high prevalence of vitamin D (94.5%), vitamin A (93.5%), vitamin B6 (60.8%), and zinc (85.6%) deficiencies. Micronutrient deficiencies among patients with cirrhosis vary by cirrhosis etiology. Reported micronutrient deficiencies among patients with alcohol-associated liver disease include vitamins B1, B2, B3, B6, B9, and B12 deficiency; vitamins A, C, D, and E deficiency; and magnesium, selenium, copper, and zinc deficiency.^5^ Adults with metabolic dysfunction–associated steatotic liver disease have reported lower intake of vitamin E and C. Adults with HCC have reported vitamin D and zinc deficiency.^5^ Due to impaired bile acid secretion and reduced fat absorption, particularly in cholestatic liver disease, fat-soluble vitamin deficiencies are also common.^5^ Our study identifies high rates of vitamin A, zinc, vitamin D, vitamin B12, vitamin E, and selenium deficiencies. Compared to the study by Llibre-Nieto et al,^1^ which was composed of predominantly hospitalized patients with alcohol-associated liver disease, the patients in our study predominantly have metabolic dysfunction–associated steatotic liver disease. Similar to the literature, zinc deficiency was significantly different across liver disease etiology, with the highest rate of zinc deficiency among patients with alcohol-associated liver disease. The rate of vitamin B6 deficiency was higher among patients with cholestatic liver disease compared to noncholestatic liver disease, but fat-soluble vitamin deficiency rates were not significantly different between the 2 groups. As only 14 patients had cholestatic liver disease in our study population, this discrepancy with the literature may reflect a small, nonrepresentative sample size. Additionally, in the geriatric literature, zinc deficiency has been described among adults with cirrhosis and has been associated with HE, frailty, and sarcopenia.^7^ Although we identified high rates of zinc deficiency among ambulatory adults with cirrhosis awaiting liver transplantation, there was no significant difference in rates of zinc deficiency between frail and not frail patients. Our study, however, did find higher rates of zinc deficiency among patients with ascites compared to those without ascites, consistent with literature findings of higher zinc deficiency rates among patients with decompensated cirrhosis and patients with cirrhosis on diuretics^1^,^12^ and by cirrhosis etiology.^5^ Overall, the vitamin deficiencies identified in our study are generally consistent with literature findings.

We found that vitamin B12, vitamin E, and selenium deficiency were independently associated with frailty among ambulatory adults with cirrhosis. These vitamin deficiencies have been described in the geriatrics literature as vitamin deficiencies also associated with frailty among older adults.

### Vitamin B12 deficiency

Vitamin B12 is associated with DNA synthesis, neurological function, and red blood cell production and is particularly seen with older age, alcohol use, use of acid-suppressing medications like proton-pump inhibitors, and surgical resection of the terminal ileum.^13^,^14^ Patients with vitamin B12 deficiency develop glossitis, pallor, cognitive impairment, gait abnormalities, peripheral neuropathy, hyperpigmentation, and impaired sense of smell. Among older adults, vitamin B12 deficiency is associated with frailty, although there is conflicting evidence on whether vitamin B12 deficiency repletion is effective in preventing frailty among older adults.^14^ Vitamin B12 deficiency leads to elevated homocysteine levels, leading to chronic inflammation, impaired DNA synthesis, and increased oxidative stress, ultimately leading to musculoskeletal dysfunction.^13^,^14^ When controlling for hemoglobin, the relationship between vitamin B12 deficiency and frailty was attenuated, suggesting partial mediation or confounding by anemia.

### Vitamin E deficiency

Vitamin E is an antioxidant that inhibits free-radical reactions and interferes with fibrosis.^6^,^15^ Patients with vitamin E deficiency develop increased platelet aggregation, hemolytic anemia, peripheral neuropathy, spinocerebellar ataxia, skeletal myopathy, and pigmented retinopathy. National guidelines recommend the use of 800 IU of vitamin E for biopsy-proven metabolic dysfunction–associated steatohepatitis without cirrhosis. In the InCHIANTI (Invecchiare in Chianti) cohort study of adults 65 years or older living in Chianti, Tuscany, Italy, vitamin E levels decreased from nonfrail to frail patients even when levels were adjusted for age and gender.^16^ Participants with higher vitamin E levels were less likely to be frail than those with lower vitamin E levels (OR: 0.30, 95% CI: 0.10–0.91).^16^ Vitamin E deficiency leads to increased oxidative stress, leading to musculoskeletal degradation. Similar to vitamin B12, when controlling for hemoglobin, the association between vitamin E deficiency and frailty was attenuated.

### Selenium deficiency

Selenium also acts as an antioxidant and is associated with thyroid hormone production and the immune response.^7^,^17^ Selenium deficiency is associated with cardiomyopathy, myositis, altered thyroid hormone metabolism, nausea/vomiting, hair loss, and skin changes. Selenium deficiency is associated with sarcopenia and frailty, and those with lower selenium intake have reduced muscle and grip strength.^17^ Similar to vitamin E, selenium deficiency also leads to increased oxidative stress, leading to musculoskeletal dysfunction.^7^,^17^ Selenium deficiency remained significantly associated with frailty when controlling for covariates, including hemoglobin level.

The association between selenium deficiency and frailty has been described in geriatrics literature.^7^,^17^ Due to widespread rates of micronutrient deficiencies described in adults with cirrhosis, the American Association for the Study of Liver Disease (AASLD) national guidelines on frailty recommend a pragmatic approach of empiric oral multivitamin supplementation among adults with cirrhosis and evidence of frailty.^5^ Future studies should evaluate whether targeted repletion of selenium deficiency is associated with improved frailty. Alternatively, given widespread multivitamin deficiency, it may be more cost-effective to empirically provide a multivitamin enriched for multiple vitamin deficiencies among all ambulatory adults with cirrhosis awaiting liver transplantation. These approaches require careful evaluation and should be considered in the context of other frailty-mitigating strategies, including dietary and exercise interventions.

This study has several limitations. First, a limited number of patients experienced adverse outcomes such as hospitalization and mortality, which limits the assessment of the association between micronutrient deficiency and clinical outcomes. Second, as a cross-sectional study, limited conclusions may be drawn on the directional nature of the association between micronutrient deficiency and frailty. Third, as micronutrient intake levels can fluctuate, particularly with events such as acute illness or recent micronutrient intake, there may be measurement bias in the data by measuring micronutrient deficiencies at only one time point. Additionally, dietary intake and prior micronutrient supplement intake may impact the measurement of micronutrient deficiencies. These data were not collected for all patients and were not included in the data analysis for this study.

Nevertheless, our study provides a contemporary evaluation of micronutrient deficiencies among ambulatory adults with cirrhosis and provides compelling evidence for the association between frailty and selenium deficiency. Reducing frailty within this population may have a profound impact on waitlist mortality and post-transplant morbidity and mortality. In addition to dietary and exercise-related frailty-mitigation strategies, future studies should assess the use of multivitamins and/or targeted evaluation of micronutrient deficiencies described as potential adjunctive therapies in this patient population.

## Supplementary Material
