# Supplementary material for: Micronutrient deficiencies in ambulatory adults with cirrhosis
Source: Hepatol Commun. 2026 Aug 11;10(9):e1032. doi: 10.1097/HC9.0000000000001032 (PMC13466105; doi:10.1097/HC9.0000000000001032)
Supplement: Supplementary file 1 [file hc9-10-e1032-s002.pdf]

Micronutrient	Lab Values	Repletion Protocol
Zinc	70 ug/dL	50mg elemental zinc/d orally
Vitamin D	Insufficiency: <30 ng/mL<br>Deficiency: <20 ng/dL	50,000 IU D2 or D3 weekly or 6000 IU daily orally for 8 weeks or until level >30 ng/ml followed by maintenance 1500-2000 IU/d orally, double or triple doses for those w/ obesity or malabsorption
Vitamin E	<5.5mg/L	400-800 IU/d orally
Vitamin B12	>0.4umol/L	1000 ug IM every other day for 3 weeks followed by 1000 ug IM monthly or 100—2000 ug/d orally
Selenium	<70mcg/L	20-100mcg IV or 50-100mcg orally per day
Vitamin A	<10 ug/dL (with concurrent vitamin E deficiency)	2000-200,000 IU/d orally
Vitamin B9	<140 ng/mL	0.4-1mg IV daily for 3d followed by 400ug/d orally
Vitamin B6	<20 nmol/L	100mg/d orally

Supplementary Figure 1. Dietitian-Driven Micronutrient Repletion Protocol Among Adults with Cirrhosis
