# Supplementary material for: Micronutrient deficiencies in ambulatory adults with cirrhosis
Source: Hepatol Commun. 2026 Aug 11;10(9):e1032. doi: 10.1097/HC9.0000000000001032 (PMC13466105; doi:10.1097/HC9.0000000000001032)
Supplement: Supplementary file 2 [file hc9-10-e1032-s001.docx]

**Supplemental Materials**

**Supplementary Figure 1. Dietitian-Driven Micronutrient Repletion Protocol Among Adults with Cirrhosis**

**Supplementary Table 1. Micronutrient Deficiencies by Cholestatic vs Non-Cholestatic Disease Status**

|  | **Non-Cholestatic Disease** | **Cholestatic Disease** | **p-value** |
| --- | --- | --- | --- |
| **Vitamin A** | 172 (75) | 10 (71) | 0.76 |
| **Vitamin B6** | 4 (2) | 2 (14) | 0.04 |
| **Vitamin B9** | 6 (3) | 0 (0) | 1.00 |
| **Vitamin B12** | 52 (22) | 3 (21) | 1.00 |
| **Vitamin D** | 152 (64) | 10 (71) | 0.78 |
| **Vitamin E** | 57 (24) | 6 (43) | 0.12 |
| **Zinc** | 170 *71) | 9 (69) | 1.00 |
| **Selenium** | 41 (17) | 3 (21) | 0.72 |

**Supplementary Table 2. Micronutrient Deficiencies by Ascites Status**

|  | **No Ascites** | **Ascites** | **p-value** |
| --- | --- | --- | --- |
| **Vitamin A** | 49 (59) | 111 (82) | <0.001 |
| **Vitamin B6** | 1 (1) | 6 (4) | 0.25 |
| **Vitamin B9** | 3 (4) | 2 (1) | 0.38 |
| **Vitamin B12** | 18 (21) | 26 (18) | 0.73 |
| **Vitamin D** | 55 (62) | 94 (66) | 0.57 |
| **Vitamin E** | 13 (15) | 36 (25) | 0.07 |
| **Zinc** | 53 (60) | 111 (79) | 0.004 |
| **Selenium** | 10 (11) | 22 (15) | 0.44 |
